# Talus Avascular Necrosis as a Rare Complication of Cushing’s Disease: A Case Report

**DOI:** 10.7759/cureus.57531

**Published:** 2024-04-03

**Authors:** Alireza Mousavian, Mohammad Abdollahi, Negin Haddadan

**Affiliations:** 1 Orthopedic Research Center, Mashhad University of Medical Sciences, Mashhad, IRN

**Keywords:** pituitary adenoma, brain tumor, ankle arthrodesis, cushing’s disease, avascular necrosis of the talus, avascular osteonecrosis

## Abstract

Avascular necrosis (AVN), also called osteonecrosis, stems from blood supply interruption to the bone and is often idiopathic. It has risk factors like trauma, alcohol, and corticosteroids. AVN in the talus (AVNT) is less common than in the femoral head. Most cases of talar osteonecrosis are associated with trauma, while a smaller proportion is linked to systemic conditions such as sickle cell disease or prolonged prednisone use. Glucocorticoids are a key nontraumatic cause. We report a middle-aged woman with Cushing’s syndrome symptoms, such as hypertension and moon face, since her youth. A few years ago, she experienced pain and swelling in her ankle, which was diagnosed as atraumatic AVNT and treated with hindfoot fusion. Years later, she was diagnosed with Cushing’s disease caused by an adrenocorticotropic hormone (ACTH)-producing pituitary adenoma in laboratory tests and imaging, which was resected in 2020. She experienced significant weight loss, and her Cushing’s syndrome symptoms were relieved after tumor resection. Mechanisms behind AVN in hypercortisolism involve fat cell hypertrophy, fat embolization, osteocyte apoptosis, and glucocorticoid-induced hypertension. Traditional X-rays may miss early AVN changes; MRI is preferred for early detection. Although there are some cases of femoral AVN caused by endogenous hypercortisolism in the literature, as far as we know, this is the first case of AVNT due to Cushing’s disease. AVNT treatment includes conservative approaches, hindfoot fusion, and core decompression. Cushing’s disease is a rare cause of AVNT, and a multidisciplinary approach aids in the rapid diagnosis of elusive symptoms.

## Introduction

Avascular necrosis (AVN), also known as osteonecrosis, is a condition arising from the temporary interruption or permanent cessation of blood supply to a bone, leading to tissue necrosis or its demise. While AVN is frequently idiopathic, certain established risk factors are known including trauma, alcohol abuse, and the use of exogenous corticosteroids [1]. While not as prevalent as in the femoral head, AVN of the talus (AVNT) in the ankle presents a painful and disabling issue for patients and poses a challenging dilemma for orthopedic surgeons [2]. About 75% of cases of talar osteonecrosis stem from traumatic injuries, while approximately 25% of nontraumatic instances are typically associated with systemic conditions such as sickle cell disease or prolonged use of prednisone, which impede blood flow. [3]

The use of glucocorticoids is one of the most important non-traumatic causes of AVN. Nevertheless, there are some case reports where AVN in the femoral head is reported as a manifestation of endogenous hypercortisolism, particularly associated with Cushing's syndrome [4-12].

In this article, we describe the case of a middle-aged woman who was diagnosed with idiopathic severe progressive AVNT for two years. She had retrogradely diagnosed masked symptoms of Cushing’s disease since her youth, but the diagnosis was confirmed after undergoing ankle arthrodesis.

## Case presentation

A 43-year-old woman visited our office in June 2018 with a complaint of severe pain and swelling in her left ankle, which had persisted for the past two years. She had hypertension since her youth and blurry vision since 2013 but had no other significant medical or family history. She was also diagnosed with major depressive disorder (MDD) in 2015 when she lost her husband. She had no history of smoking, alcohol consumption, or addiction. She had not experienced any significant trauma during this period and sought consultations from various specialties, including neurology, psychology, internal medicine, nephrology, rheumatology, and orthopedics. She had received a platelet-rich plasma (PRP) injection in the ankle, but it did not improve her symptoms. Despite undergoing various diagnostic workups, no precise diagnosis had been established.

Back in 2013, she remembers suddenly experiencing blurry vision in her right eye. This condition underwent a misdiagnosis, which almost led to a loss of vision. She had been struggling with her eye problems until her last visit, during which she received intravitreal bevacizumab injections. Additionally, she previously had iron deficiency anemia, which was treated with ferrous sulfate before 2018.

**Figure 1:**
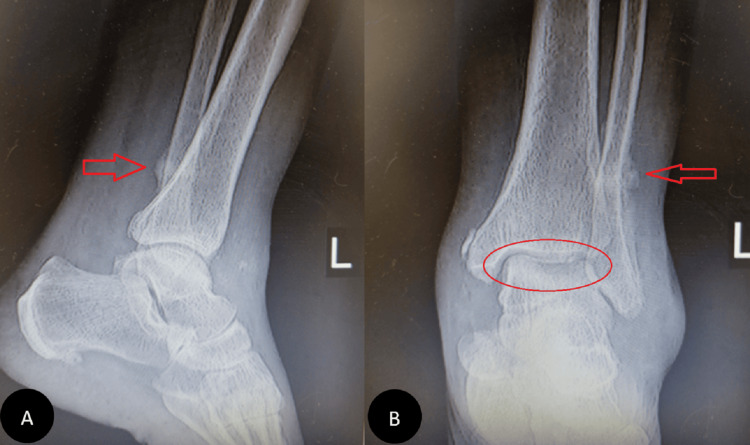
Ankle X-ray six months after arthroscopy Pain had reduced for four months, then pain increased with activity and disabled her after a night of dancing. Subchondral fracture and fibular stress fracture are evident (A and B, respectively).

**Figure 2:**
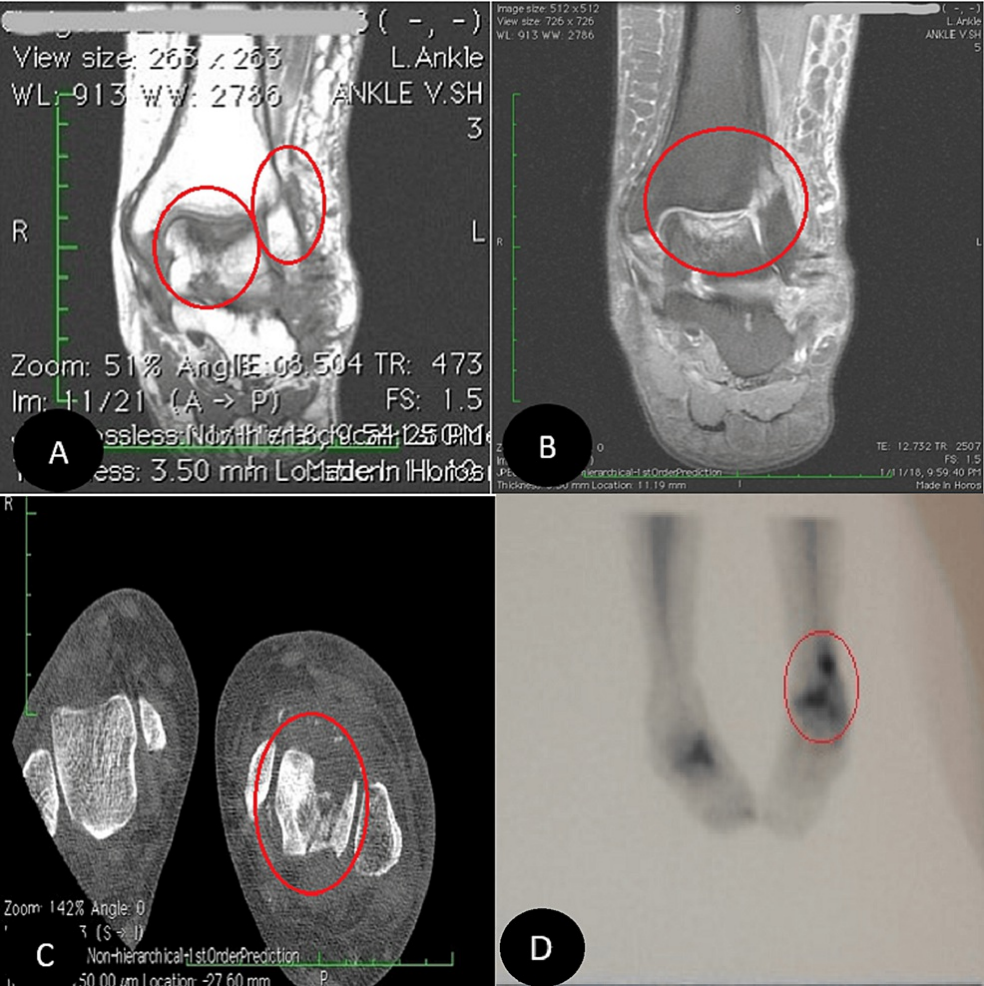
MRI, CT scan, and technetium-99m (Tc-99m) bone scan Coronal MRI confirmed avascular necrosis of the talar dome with subchondral fracture (A and B, respectively). CT scan (C) and Tc-99 bone scan (D) images also revealed the pathologies.

In our first visit, during the physical examination, the pain was localized in the ankle mortise with some posterolateral pain along the course of the peroneal tendons posterior to the fibula. Based on the physical examination and available ankle radiographs, we diagnosed AVNT. The patient subsequently underwent ankle arthroscopy through the standard anterior portals, the joint was cleaned, the synovium was shaved, and a small incision was conducted for peroneal assessment; this procedure revealed a subchondral collapse and extensive necrosis in the talus. Following the procedure, she experienced a partial improvement in her symptoms. However, six months later, she returned with a recurrence of symptoms (Figure 1). Upon further inquiry, she mentioned that her symptoms had recurred a month ago when she was dancing at a family party. Radiographs showed a stress fracture in her fibula and extensive AVNT. This diagnosis was confirmed through a CT scan, MRI, and bone scan (Figure 2).

**Figure 3:**
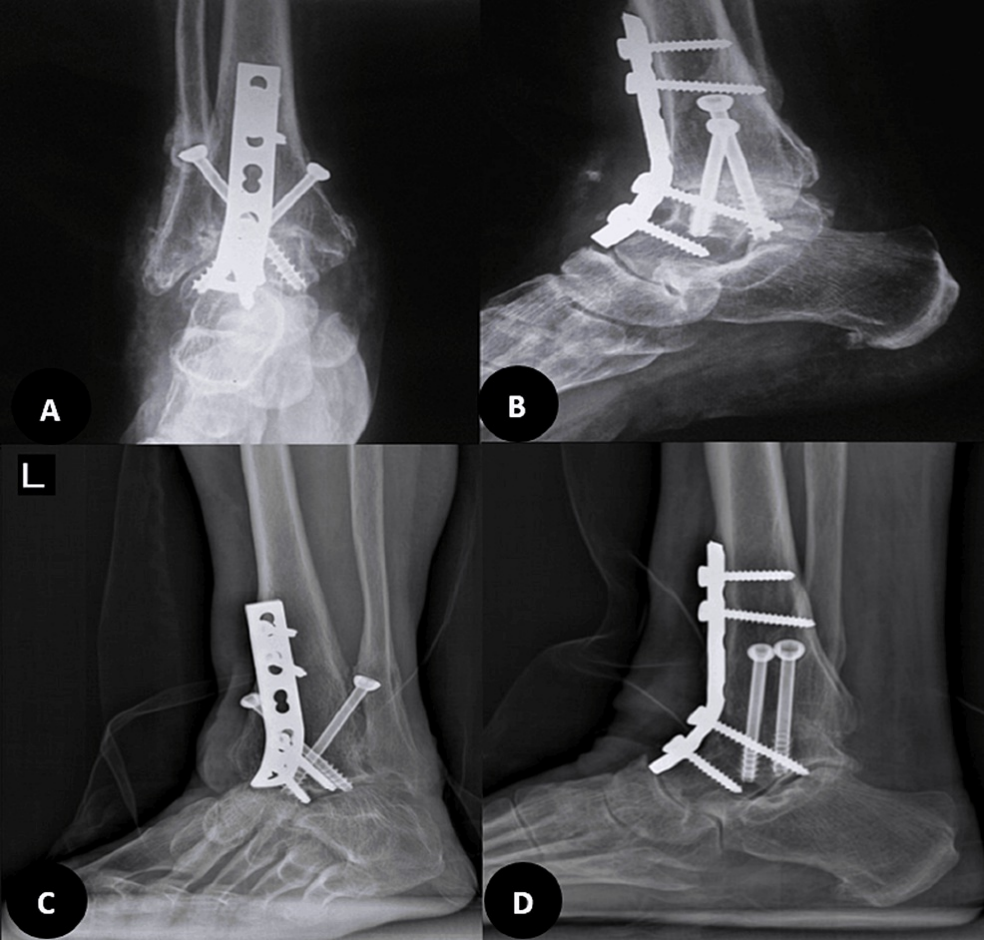
Post-operative radiographies Six months after the ankle surgery, a huge osteonecrosis and fibular stress fracture were managed with an acceptable, painless ankle fusion (not solid) despite the remaining necrosis (A and B, respectively). In 2024, four years after the tumor resection, complete healing of talus necrosis and solid fusion were achieved (C and D, respectively).

In the second visit after arthroscopy, upon confirmation of a fibular stress fracture and significant subchondral collapse, and following a discussion of the next available options with the patient, the second procedure was performed as an ankle arthrodesis with an anterior approach. A 6 cm longitudinal incision was made anteriorly, and through the plane between the tibialis anterior and extensor hallucis longus, the ankle joint was accessed. Joint preparation was done with an osteotome, ensuring a bleeding surface on both sides. Then, manual compression with provisional pin fixation in the corrective position was performed. The fusion was planned at less than 5 degrees of valgus, 10 degrees of external rotation, and approximately 10 degrees of plantar flexion, suitable for the high-heeled shoes that she was using in her daily living activities. After confirming fluoroscopy in two planes, final 6.5 mm cannulated cancellous screws were used, and fixation was augmented with an anterior molded 4.5 mm narrow dynamic compression plate (DCP), according to our previously published anterior ankle fusion technique [13]. The foot was placed in a splint for 10 days, after which stitches were removed, and a cast was applied for four weeks. Then, walking with gradual, as-tolerated weight-bearing was initiated (Figure 3). Three months after surgery, she was pain-free, and by the sixth month, she could walk without any boot or brace, only using high-heeled shoes.

**Figure 4:**
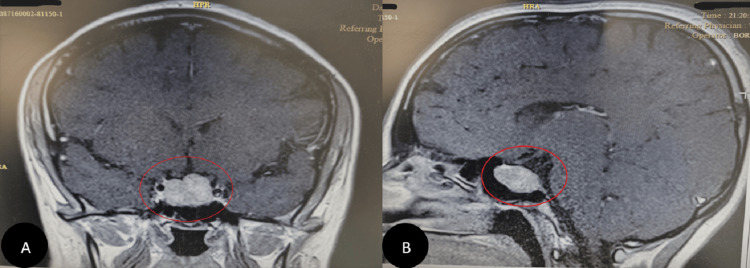
Brain MRI Finally, a pituitary adenoma was diagnosed using a Brain MRI as the cause of Cushing’s disease symptoms (A and B).

In 2020, two years after her ankle surgery, she was referred to an endocrinologist due to excessive weight gain and hirsutism. The biochemical assessment revealed the following: cortisol (8 AM) (chemiluminescence immunoassay (CLIA)) was 96 µg/dl (normal range: 4.82 - 19.5 µg/dl), and it was 22.1 µg/dl after overnight dexamethasone (normal range: < 1.8 µg/dl). Adrenocorticotropic hormone (ACTH) (CLIA) was 44.4 pg/ml (normal range: 7.2-63.3 pg/ml), and cortisol measured 5.7 µg/dl after the 48-hour low-dose dexamethasone suppression test (normal < 5 µg/dl). The results, along with symptoms (Table 1), are documented in the laboratory tests (Table 2). She was diagnosed with Cushing’s syndrome, which was subsequently confirmed as Cushing's disease due to an ACTH-producing pituitary adenoma observed in the MRI and Brain CT (Figure 4).

**Table 1:** Cushing's disease symptoms and signs The hyphens in the table indicate that the patient does not have those symptoms or signs.

Sign/symptom	Severity
Weight Gain	Severe
Hirsutism	Severe
Hypertension	Severe
Easy bruising	Severe
Depression	Severe
Moon face	Moderate (masked with makeup)
Lethargy	Moderate
Headache	Moderate
Peripheral edema	_
Buffalo hump	_
Myopathy	_
Acne	_
Purple striae	_

**Table 2:** Laboratory tests CLIA: chemiluminescence immunoassay; ACTH: adrenocorticotropic hormone; LDDST: low-dose dexamethasone suppression test

Laboratory test	Result	Reference range
Cortisol (8 AM) (CLIA)	96 µg/dl	4.82-19.5 µg/dl
Cortisol (8 AM) (after overnight dexamethasone) (CLIA)	22.1 µg/dl	<1.8 µg/dl
ACTH (CLIA)	44.4 pg/ml	7.2-63.3 pg/ml
Cortisol after 48 hours of LDDST (CLIA)	5.7 µg/dl	< 5 µg/dl

Finally, she underwent a tumor resection and had a dramatic response after treatment (30 kg weight loss). She revealed that she had Cushing’s syndrome symptoms since she was young. These symptoms included a puffy face, which she covered with makeup, high blood pressure, and hirsutism. In January 2024, four years after her brain surgery, during our last visit, her symptoms had significantly improved. She reported no problems with her ankle, and talus necrosis was completely healed, with a solid fusion achieved in radiographs (Figure 3).

## Discussion

As far as we are aware, this case presentation represents the first instance of AVNT attributed to Cushing’s disease in the existing literature. Nevertheless, some individuals with endogenous Cushing's syndrome have been reported to experience AVN of the femoral head [4-12].

Cushing's syndrome is an uncommon endocrine condition marked by manifestations of hypercortisolism. The predominant cause is often an adenoma in the anterior pituitary gland that produces ACTH, referred to as Cushing's disease [14]. The presentation of Cushing's syndrome can vary significantly in both adults and children, influenced by the extent and duration of hypercortisolemia. However, the typical signs and symptoms of Cushing's syndrome are widely known [15]. Although some individuals may perceive these alterations as normal and physiological, the disease can go unnoticed for an extended period, as in our case, in which it remained undiagnosed for more than 20 years.

However, it is known that steroid use is a significant contributing factor to the occurrence of bone osteonecrosis, accounting for up to 40% of non-traumatic instances of AVN [16]. The mechanisms leading to AVN due to either endogenous hypercortisolism or excess exogenous glucocorticoids are not completely understood. There are just some hypotheses that suggest that the hypertrophy of fat cells, embolization of fat, and osteocytes' apoptosis result in impaired blood flow in the bone, ultimately causing ischemic tissue necrosis [17]. An alternative proposed theory suggests that elevated levels of glucocorticoids may cause insulin resistance and subsequently contribute to AVN [18].

Traditional X-rays often fail to detect the initial changes of AVN (as observed in our case). MRI stands as the preferred method for identifying AVN in its early phases, providing an opportunity for timely therapeutic interventions [19,20].

In an analysis of 321 cases of AVNT, the predominant treatment modalities included conservative therapies (n = 104), hindfoot fusion (n = 62), and core decompression (n = 85) [21]. These approaches reflect the primary methods employed in contemporary clinical practice for addressing AVNT.

**Figure 5:**
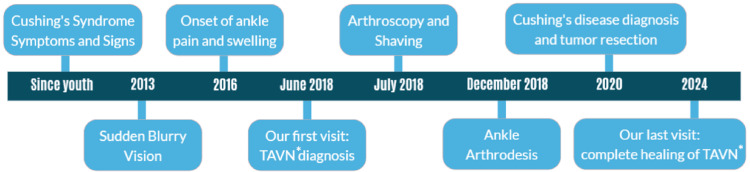
Case report timeline * Avascular necrosis in the talus

After all, we confirmed the AVNT diagnosis using MRI and bone scan and managed it with hindfoot fusion. Subsequently, the underlying issue, endogenous hypercortisolism due to an ACTH-producing pituitary adenoma, was identified and treated through resection of the tumor (Figure 5).

## Conclusions

Cushing’s syndrome is a rare endocrine disorder characterized by excessive cortisol levels, commonly caused by an ACTH-producing adenoma in the pituitary gland, known as Cushing’s disease. Cushing’s disease may be one of the rare causes of AVNT. To the best of our knowledge, this is the first instance of AVNT due to Cushing’s disease described in the literature. Since atraumatic AVNT is rare in itself, a multidisciplinary approach can lead us to a more rapid and proper diagnosis, as each symptom may be masked or considered rare within its subspecialty field.
